# Deciphering cellular states of innate tumor drug responses

**DOI:** 10.1186/gb-2006-7-3-r19

**Published:** 2006-03-15

**Authors:** Esther Graudens, Virginie Boulanger, Cindy Mollard, Régine Mariage-Samson, Xavier Barlet, Guilaine Grémy, Christine Couillault, Malika Lajémi, Dominique Piatier-Tonneau, Patrick Zaborski, Eric Eveno, Charles Auffray, Sandrine Imbeaud

**Affiliations:** 1 Array s/IMAGE, Genexpress, Functional Genomics and Systems Biology for Health, LGN - UMR 7091 - CNRS and Pierre and Marie Curie University - Paris VI, Villejuif, France

## Abstract

**Background:**

The molecular mechanisms underlying innate tumor drug resistance, a major obstacle to successful cancer therapy, remain poorly understood. In colorectal cancer (CRC), molecular studies have focused on drug-selected tumor cell lines or individual candidate genes using samples derived from patients already treated with drugs, so that very little data are available prior to drug treatment.

**Results:**

Transcriptional profiles of clinical samples collected from CRC patients prior to their exposure to a combined chemotherapy of folinic acid, 5-fluorouracil and irinotecan were established using microarrays. Vigilant experimental design, power simulations and robust statistics were used to restrain the rates of false negative and false positive hybridizations, allowing successful discrimination between drug resistance and sensitivity states with restricted sampling. A list of 679 genes was established that intrinsically differentiates, for the first time prior to drug exposure, subsequently diagnosed chemo-sensitive and resistant patients. Independent biological validation performed through quantitative PCR confirmed the expression pattern on two additional patients. Careful annotation of interconnected functional networks provided a unique representation of the cellular states underlying drug responses.

**Conclusion:**

Molecular interaction networks are described that provide a solid foundation on which to anchor working hypotheses about mechanisms underlying *in vivo *innate tumor drug responses. These broad-spectrum cellular signatures represent a starting point from which by-pass chemotherapy schemes, targeting simultaneously several of the molecular mechanisms involved, may be developed for critical therapeutic intervention in CRC patients. The demonstrated power of this research strategy makes it generally applicable to other physiological and pathological situations.

## Abstract 2

Transcriptional profiling of colorectal cancer samples collected before chemotherapy provides cellular signatures that differentiate subsequently diagnosed chemosensitive and resistant patients.

## Background

Colorectal cancer (CRC) remains a leading cause of cancer death in the Western world, although cancer therapy has benefited from promising new compounds directed to various targets [1-3]. For instance, 5-fluorouracil (5-FU)-based chemotherapy, usually biomodulated with low-dose leucovorin (folinic acid (FA)) has been used widely for over 40 years, resulting in a reduction of cancer recurrence and associated death rate [4,5]. In the mid 1990s, CPT-11 (Campto^®^, irinotecan, 8-ethyl-10-[4(1-piperidino)-1-piper-idino] carbonyloxy camptothecin) was described as one of the most active drugs and a promising anti-cancer agent [6,7].

A major obstacle to successful treatment arises from the emergence of states of resistance to anti-cancer drugs, preventing elimination of metastatic cells. Some clinical studies proposed combined chemotherapy to leverage the efficiencies of different drugs by attacking simultaneously distinct biochemical targets for the purpose of overcoming drug resistance in heterogeneous tumors. Thus far, some key regimens such as FOLFIRI (folinic acid, 5-FU, irinotecan) have demonstrated tolerable side effects and a broad spectrum of efficacy against solid tumors. The use of FOLFIRI has resulted in prolonged survival and improved response rates. Nevertheless, emergence of clinical drug resistance remains the primary cause of failure during chemotherapeutic treatment of most human tumors [8,9]. Human cancers are mostly found to be resistant to therapy at the time of drug presentation (primary responses), tumors being intrinsically drug resistant (innate or *de novo *drug resistance). Only a few become resistant after an initial response (acquired responses), the tumors developing resistance to chemotherapy during treatment (acquired drug resistance), reviewed in [10,11]. In the latter group, a tumor cell may express drug resistance by combining several distinct mechanisms induced by its exposure to various drugs. In the former group, however, this is unlikely to be the case.

Biochemical and histo-pathological technologies have been applied in order to identify relevant mechanisms that may have important implications for drug efficacy and actively contribute to innate resistance. Thus, high levels of thymidylate synthase, the 5-FU target, were found associated with tumor insensitivity to FU-based therapy [12,13]. Similarly, higher levels of topoisomerase-I (TOP1) correlated with greater sensitivity of colon tumors to camptothecin derivatives compared to normal colonic mucosa [14]. Glucuronidation, involved in xenobiotic detoxification, was also shown to be associated with innate resistance to TOP1 inhibitors in colon cell lines and tumors [15,16]. Finally, an increase of the ABCB1/P-gp transporter, a member of the family of ABC-transporters that detect and eject anti-cancer drugs from cells, was observed in intrinsically drug-resistant colon tumors [17].

Studies of cancer cell lines have emphasized an array of potentially general mechanisms of drug resistance. Expression of BCL2 obtained by transfection in a human lymphoma cell line rendered it resistant to FU-mediated apoptosis [18], while p53 was found over-expressed in human embryo fibroblasts resistant to FU-mediated growth inhibition [19], and protein kinase C in colon adenocarcinoma cell lines resistant to doxorubicin [20,21]. In addition, over-expression of specific drug transporters (ABCB1/P-gp, LRP (lung resistance-related protein) or MRP (multidrug resistance-related protein)) was shown by flow cytometry and fluorescence microscopy to occur in human colon adenocarcinoma cell lines resistant to TOP1 inhibitors [20,22].

Our current understanding of mechanisms associated with drug resistance has been furthered by investigating drug-resistant cellular models created by exposing a parental population (yeast, bacteria, mammalian cell lines) to increasing concentrations of a cytotoxic agent [23-26]. It has been difficult, however, to translate these insights into clinically meaningful improvements in cancer treatment, suggesting that *in vitro *unicellular models may not be applicable to the *in vivo *situation or represent the disease in its entirety. For instance, in CRC, *TOP1 *mutations that decrease the formation of DNA cleavage complexes were identified [27], but their implication in clinical resistance was not confirmed.

Since the introduction of molecular genetics methods in clinical oncology, examination of individual mRNA/protein expression levels of drug target molecules provided complementary indications on the mechanisms involved. Thus far, however, only a limited number of clinical studies of drug resistance have focused on individual candidate genes and these used clinical samples exclusively derived from patients that were already treated with drugs. In CRC, such gene-by-gene molecular biology studies have highlighted only a partial list of candidate genes [28-33]; some of these genes were shown to be involved in mechanisms altering drug metabolite potency, others are known to participate in increase of drug efflux or decrease of drug toxicity, or to participate in inhibition of apoptosis (for an overview, see [32-37]). It is unclear at present whether these mechanisms play a causative role in clinical drug resistance, and no comprehensive analysis of entire drug resistance pathways has been conducted.

Pharmacogenetics and pharmacogenomics approaches have been initiated to study the relationship between individual variations and drug response rates [38,39]. Genetic polymorphisms of specific genes were found to be associated with clinical outcomes in patients treated through chemotherapy, and amplification of genes encoding drug targets or transporters was shown to alter the sensitivity of cancer cells to a particular chemotherapy [40,41]. Finally, loss of heterozygosity at specific regions of chromosomes was identified in specific carcinoma, although its consequence in treatment outcome remains controversial [40,41].

High-throughput gene expression profiling with microarrays has been introduced recently in some cancer clinical studies, with the potential to provide a genome-wide coverage of the molecular pathways involved in cancer development and treatment. These studies, however, have not directly addressed the issue of drug resistance but rather have compared tissue sample phenotypes (normal versus tumor) or developmental stages of the disease for the purpose of tumor classification [42-48].

Common to all of these studies is the fact that the models investigated do not address mechanisms that contribute to innate drug resistance, but rather test hypotheses on how drug exposure induce resistance states. In addition, it is not known whether drug resistance mechanisms identified after drug exposure (acquired drug resistance) are relevant to tumor cell survival occurring after initial drug treatment (innate drug resistance). Previous reports proposed that a specific tumor microenvironment may provide a sanctuary for subpopulations of tumor cells that gain a survival advantage following initial drug exposure, and that this may facilitate emergence of acquired drug resistance [49]. In sum, while there are many possible assumptions, much work remains to be done to provide a solid foundation on which to anchor working hypotheses about underlying mechanisms of clinical (*in vivo*) tumor drug resistance.

Here, we report the results of the proof-of-concept initial phase of a biological systems approach [50] toward understanding innate CRC tumor responses to a FOLFIRI combined chemotherapy of irinotecan (CPT-11) plus 5-FU/FA. Gene expression patterns obtained with microarrays were compared between clinical samples from colon tumors and liver metastases collected from CRC patients prior to drug exposure. We illustrate how the use of a vigilant experimental design, power simulations and a robust statistical analysis allowed us to restrain the false negative and positive differential hybridization rates to a minimum [51]. We integrated the data collected from a biological systems perspective into global and interconnected molecular networks that highlighted the molecular mechanisms that may anticipate resistance in CRC patients prior to their exposure to drugs, and discuss how this knowledge could be used in clinical practice as a complement to clinical, biochemical and genetic markers for global prevention, early diagnosis and better patient treatment.

## Results and discussion

### Biological variability and similarity in gene expression profiles

Gene expression profiles were collected on colon tumor (T), adjacent non-tumoral colon (N) and liver metastasis (M) samples surgically resected from CRC patients prior to their exposure to a combined chemotherapy. A total of 26 RNA samples from 13 patients were included in the workflow (see Additional data file 1), following a vigilant quality-control procedure performed at 3 levels of qualification: RNA quality, linear amplification and target synthesis specifications (for detailed procedures, see Additional data file 2). RNA samples were dye-labeled and hybridized in quadruplicate to a human microarray, ensuring genome-wide coverage of functional pathways and networks, as described in Material and methods and Additional data file 2. The resulting 3.2 × 10^6 ^hybridization data points collected from 70 arrays were stored in a database and preprocessed for normalization and filtering.

**Figure 1:**
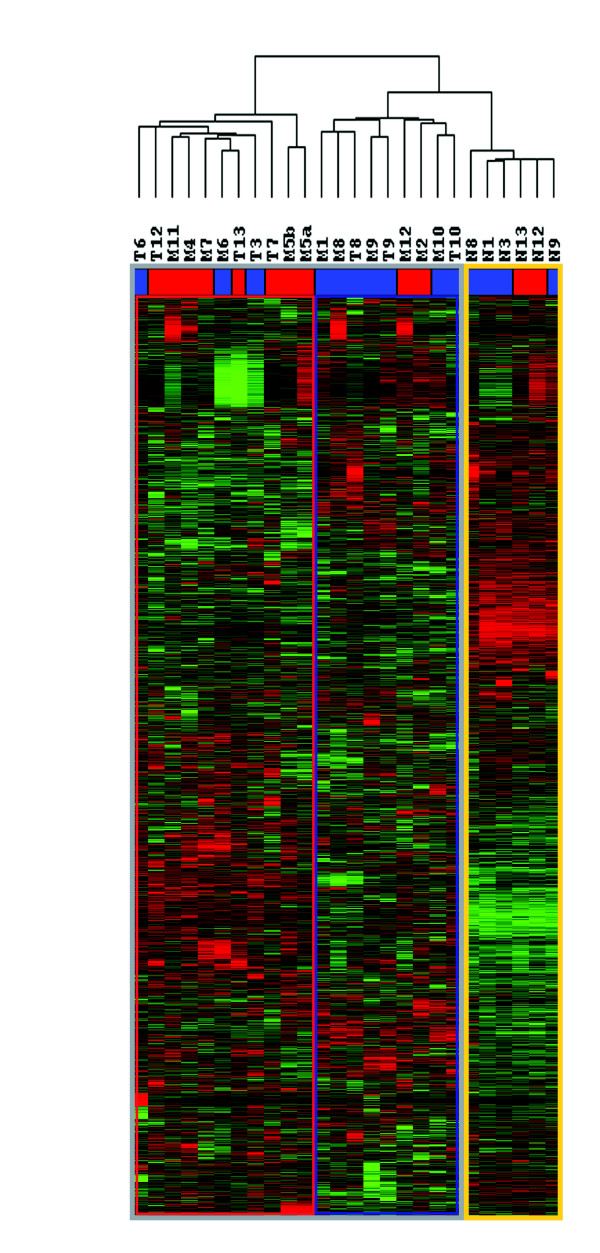
Range of biological variability of the gene expression dataset. Similarity dendrograms (Pearson correlation) resulting from hierarchical clustering of tumor samples from CRC patients based on the global gene expression matrix. Letters refer to the origin of the biopsies, colon tumors (T), liver metastases (M) or adjacent non-tumoral colons (N). Numbers refer to individual patients. For each sample, the color patch represents the primary drug response rate, either resistant (in red) or sensitive (in blue), subsequently diagnosed in patients at the presentation of the drugs. Color squares around the gene expression matrix display specific subgroups of clusters. The yellow square shows the cluster of N samples, while the gray one refers to the clusters of cancerous samples (T&M); red and blue squares specify predictive (resistant and sensitive) subgroups of cancerous samples.

The hybridization signals registered on individual arrays followed a normal distribution and were thus suitable for statistical analysis for assessment of the range of biological variability and similarity in expression profiles (data not shown). An average linkage hierarchical clustering of tissue samples was performed using the expression profiles of all genes, revealing much structure in the data. In Figure 1, relatively high expression levels are indicated in red and relatively low expression levels in green, with each column representing data from a single tissue sample, and each row the series of measurements for a single gene. Tissue samples or genes with similar expression patterns are thus clustered adjacent to one another.

Examination of the expression matrix indicates that samples of the same origin cluster in discrete groups, with adjacent non-tumoral colon samples forming one group (N, yellow square in Figure 1), and tumor samples (T or M, gray square in Figure 1) another. Clustering of the non-tumoral samples appeared to be triggered by groups of up- or down-regulated gene expressions that are lost in the tumor samples. The Pearson correlation distance (*r*) was used as a metric of similarity, indicating higher similarity within each group of samples (N, *r *= 0.88; T, *r *= 0.69; M, *r *= 0.55) than between the different groups (N&T&M, *r *< 0.45). In addition, serial (tumor and metastasis) tumor samples obtained from individual patients appeared to cluster together, indicating that changes in expression profiles occurring during the metastatic process are less prominent than individual biological variation when measured on a genome-wide scale.

The lack of a clear distinction in expression profiles between colon tumors (T) and liver metastases (M) is striking. Both types of tumor samples occupy adjacent branches in Figure 1, and are almost as similar when grouped together (T&M, *r *= 0.56) as each subgroup considered separately (T, *r *= 0.69; M, *r *= 0.55), indicating that their relatively uniform gene expression matrix reflects complementary measurements for similar biological conditions. Furthermore, the samples from patients subsequently diagnosed as chemo-sensitive (labeled blue in Figure 1) and resistant (labeled red) exhibit an overall tendency to form clustered subgroups, despite a small number of interspersed tumor samples; their gene expression matrix was found to branch into two fairly predictive subgroups of 9 and 11 tumor samples (blue and red squares in Figure 1), in which only 2 (M2 and M12) and 3 (T3, T6 and M6) samples cluster in the alternative group. These data suggest that specific differences in the global gene expression patterns recorded might be associated with functional states and molecular mechanisms that are intrinsically causal of the two types of innate drug responses. The likelihood of detecting these specific changes is dependent on the precision of the data recorded, ensured here by an experimental design including an appropriate level of replication, and on the statistical power of the dataset to distinguish the biological conditions investigated.

### Statistical precision and power of the gene expression dataset

The statistical power of a gene expression dataset is the probability of obtaining statistical significance when true biological differences exist between the groups compared (1 - β; true positive rate). It allows verifying which subgroups of samples are likely to provide the most comprehensive relevant information and that enough samples are compared to meet the objectives of the study. A conventional statistical power analysis requires specification of parameters such as anticipated variability of individual measurements for genes within each biological group (σ), sample size (n), magnitude of the effect to be detected (Φ) and acceptable false positive rate (significance level α).

In previous experiments addressing the question of *in vitro *innate drug resistances with cellular models [24], sample coefficients of variation (CVs (standard deviation/mean) × 100), which estimate variance due to biological replicates, were reported to be under 0.4 (CVs < 40%) and the number of truly differentiated genes over 400. The distribution of the standard deviations among biological replicates was evaluated for each gene and each group of samples (N, T, M, T&M) in the recorded dataset. For 75% of the genes and the entire set of samples, CVs were below 27% (N, 19%; T, 22%; M, 27%; T&M, 27%).

Statistical power simulations were performed for two-class comparison statistics (see formulae in Additional data file 2). The expected statistical power (1-β) for detecting a true 2-fold mean difference between two groups (Φ = 1, with base 2 logarithm) at a significance level (α) of 0.003, which accounts for less than 33 false positives in the 11K microarray, and σ = 0.4 as reported in previous experiments was estimated to be 0.09, 0.27 and 0.70 for the T, M and T&M groups of comparison, respectively, and over 0.40, 0.67 and 0.98, respectively, when using σ = 0.27 as measured in the recorded dataset.

These power simulations suggest that the T&M group, which displays a relatively uniform gene expression matrix, appears likely to provide the most comprehensive information relevant to the chemo-sensitive and resistant states. Thus, even with the limited number of individual samples available, the theoretical statistical power of our recorded dataset appears to be satisfactory as it is consistent with a small number of spurious discoveries (α = 0.003), while limiting the proportion of false negatives (β = 0.02 to 0.3). Complete descriptive statistical power simulations are summarized in Additional data file 3.

### Identification of genes involved in innate drug responses

**Figure 2:**
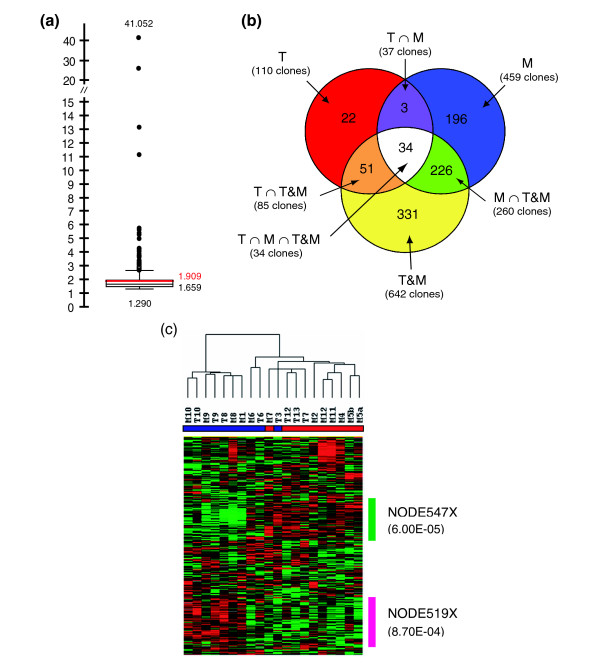
Differential gene expression in cancerous samples. **(a) **Box plot of fold changes in expression levels from genes in list L863. A red line represents the average; a black line, the median. The 1st quartiles, 3rd quartiles, minimum and maximum values are indicated; the ratios are shown in absolute values. **(b) **Venn diagram representation of the co-occurring genes with differential expressions computed through statistical comparisons of subgroups of colon tumors (T), liver metastases (M) or both cancerous tissues together (T&M) (α = 0.01). The number of clones representing genes that were found significantly differentially expressed between chemo-sensitive and resistant states is indicated. **(c) **Clustering analysis on cancerous samples of the expression profiles of the genes in list L863. Genes (row) and samples (columns) are clustered independently using Pearson correlation. The top color patch represents primary drug responses, either chemo-sensitive (in blue) or resistant (in red). The top-ranked relevant gene clusters selected using *t* statistics with permutation-based adjustment (*n* = 10,000; α = 0.05) are indicated by color bars.

To identify the genes that may be linked to innate drug responses, we estimated the number of genes that are specifically up- or down- regulated in tumors. A comparison of mean relative expression levels was performed gene-by-gene between chemo-sensitive and resistant subgroups of T or M, considering the initial response rates subsequently diagnosed for the corresponding patients. The subgroups of T&M were also compared independently. A combination of *z-*statistics together with false discovery rate (FDR) corrections to compensate for multiple testing effects, at a significance level (*α*) of 0.01, yielded a total of 863 clones (L863), corresponding to 7.5% of the clones represented on the array (Figure 2). Differential hybridizations range from 1.3- to 41-fold changes, with an average of a 1.7-fold change and about half ≥ 2-fold changes (Figure 2a).

Using Unigene Cluster IDs from build 184 (June 2005) as the common identifiers, the clones in L863 correspond to 795 unique genes, including 679 named genes and 116 sequences with no associated identifier (no ID). Among the 515 named genes found differentially expressed with greater than 1.5-fold changes, 253 showed higher and 262 lower expression in the resistant samples, while 84 over the 116 sequences with no ID displayed a similar range of differential expression, one-third being up-regulated. Finally, a small fraction of the genes (5%) showed ≥ 3-fold changes in expression levels. The number of co-occurring genes with differential expression detected in the T, M and T&M groups of samples is summarized in a Venn diagram displayed in Figure 2b. Descriptive statistics and annotations of L863 are shown in Additional data file 4.

To further probe the ability of different subsets of the genes represented in L863 to discriminate the chemo-sensitive and resistant states on a co-regulation basis, hierarchical clustering of the expression profiles was performed (Figure 2c). Clusters of gene modules that appeared most relevant to differentiate chemo-sensitive and resistant subgroups of samples were identified using *t* statistics with permutation-based adjustment (*n* = 10,000) of the gene expression matrix, α = 0.05. The top-ranked clusters (Additional data files 5 and 6) were NODE519X of 118 clones (86 named genes and 22 with no ID) found up-regulated in the chemo-sensitive subgroup of tumor samples (*t* stat = 3.53; *p* = 8.7e-04) and NODE547X of 102 clones (83 named genes and 12 with no ID), which conversely represents a cluster of genes more intensively expressed in the resistant subgroup of tumor samples (*t* stat = -4.77; *p* = 6e-05).

### Validation of the microarray gene expression data

The accuracy and reliability of the results obtained with microarrays was tested by quantitative RT-PCR (Q-PCR); using non-amplified total RNA provided a means to assess a possible bias introduced during the T7 amplification step used in the microarray analysis. The gene expression levels obtained by Q-PCR were normalized to that of the glucuronidase beta (*GUSB*) housekeeping gene and expressed as the fold increase or decrease relative to that of a referential median of the values observed in colon tumors or liver metastases, respectively, as described in Material and methods.

Twenty-two genes were chosen from list L863, and eight more were selected as not significantly differentially expressed (Table 1). First, the correspondence of expression levels in both groups of samples (T and M) was investigated and confirmed expected similarities for over 77% of the genes, with very small variances (accounting for less than one threshold cycle (Ct); Table 2). Then, Q-PCR results were compared to those obtained in the microarray hybridizations using the same samples. A good agreement was observed between microarray and Q-PCR analyses using *z*-statistics with FDR adjustments, with 22 of the 30 genes fully validated and 2 more found significantly differentially expressed in colon tumors but not in liver metastases (*α *= 0.05; Table 2). Discordant results were obtained for the remaining six genes, representing probable false negatives or false positives of one or the other technology (Table 2). Thus, differential expression detected by microarray analysis is highly predictive of expression levels measured with an independent methodology such as Q-PCR (>80% confirmation).

**Figure 3:**
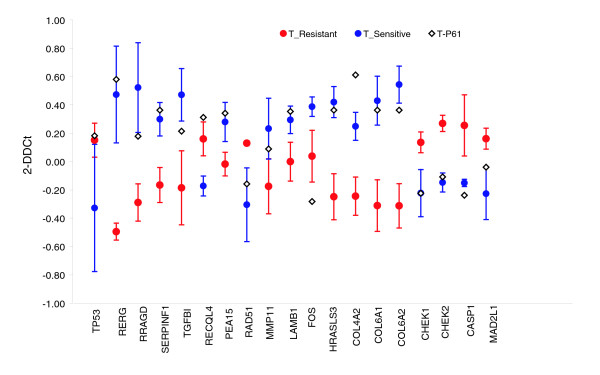
Independent biological validation by Q-PCR. Serial representation of Q-PCR relative expression (2^-ΔΔCt^; log10) of a series of genes (identified by symbols and HUGO nomenclature) that were shown to be statistically differentially expressed in both microarray and Q-PCR (indicated in bold in Table 2). Results are displayed as circles for the mean 2^-ΔΔCt ^and 95% confidence intervals (α = 0.05) computed on a series of cancerous samples, which correspond to the sample set previously used in microarray analysis (compare Additional data file 10). The color patch refers to the primary drug responses, either chemo-sensitive (in blue) or resistant (in red) of the corresponding patients. Diamonds show the relative expression levels measured on a new tumor sample (T-P61) from an additional patient (P61). The results obtained for a second new patient are presented in Additional data file 7.

For a second level of validation, two new cancer samples - one colon tumor and one liver metastasis - collected from additional patients (T-P61 and M-P52, respectively), were used for independent biological validation by Q-PCR on the same gene set. The results were compared to the relative expression levels collected on the previous sample set, focusing on statistically significant differential expression detected by both technologies (Table 2, indicated in bold). The gene expression levels observed in the new samples were found to be mostly predictive of a chemo-sensitive state (>84% confirmation), in agreement with the fact that both patients were subsequently diagnosed as sensitive upon exposure to the drugs (Figure 3 and Additional data file 7). Altogether, these results verified the reliability and the rationality of our strategy to identify genes that are commonly differentially expressed in innate drug resistance. This suggests that the genes represented in L863 could be used for further validation of drug response prediction using a larger patient cohort. These genes also represent valuable targets of further biological studies aimed at deciphering the underlying molecular pathways.

### Enrichment of the selected genes in Gene Ontology terms

**Figure 4:**
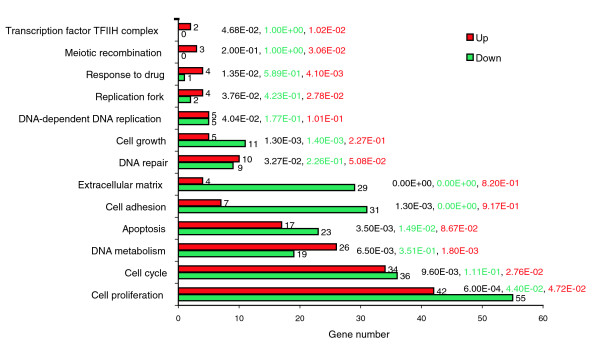
Term-ranking of GO categories. Representation of the 13 top-ranked functional categories (terms), using GO terms, that are enriched in differentially expressed genes (α = 0.05). Numbers of genes per category are indicated. The computed two-sided Fisher's exact scores are shown for each relevant biological theme (black) together with scores for up-regulated (red) or down-regulated (green) genes.

Searches were made for significantly enriched gene classes in list L863, as defined by Gene Ontology (GO) annotation associations [52]. The *p* values for the classes most enriched in list L863 appeared highly significant over what would be expected by chance (α = 0.05). First, with a one-sided jackknife Fisher's exact probability test, using the over-representation function of EASE online [53], 147 GO terms were found significantly enriched in modulated genes (*n* = 3). The 'Extracellular matrix' term achieved the highest degree of significance (*p *= 4.38e-07). Then, two-sided Fisher's exact probabilities were computed with the GoMiner software [54], identifying significant enrichment within gene classes and accounting for up-regulations (41%), down-regulations (45%) or equally balanced changes (14%). Figure 4 shows the differential expressions (up, down) from part of the top-ranking biological terms (α = 0.05).

The significantly enriched gene classes found up-regulated in resistant versus chemo-sensitive states are highly indicative of processes of cell division and DNA metabolism. They are associated with terms such as 'Replication forks' (6 genes), 'Cell cycle' (70 genes), 'M phase' (17 genes), 'Response to drug' (5 genes), 'DNA metabolism' (45 genes), 'Response to DNA damage stimulus' (26 genes) and 'DNA repair' (19 genes). Conversely, the significantly enriched gene classes that were down-regulated in resistant versus chemo-sensitive states were highly indicative of processes involving cell proliferation/adhesion and extracellular matrix (ECM) constituents and organization. The prominent terms associated with these classes are 'Cell death' (42 genes), 'Apoptosis' (40 genes), 'Cell proliferation' (97 genes), 'Cell growth' (16 genes), 'Cell motility' (27 genes), 'Cell adhesion' (38 genes), 'Cell communication' (161 genes), 'Regulation of cell cycle' (44 genes) and 'Extracellular matrix' (33 genes). Complete lists of enriched GO terms and expression changes are available in Additional data file 8. Interestingly, both NODE519X and NODE547X, described in previous sections, were enriched in the above gene classes, and associated with the 'Extracellular matrix', 'Cell adhesion' and 'Cell growth' terms (NODE519X) and the 'Cell cycle', 'Cell proliferation', 'Response to DNA damage stimulus' and 'DNA repair' terms (NODE547X).

**Figure 5:**
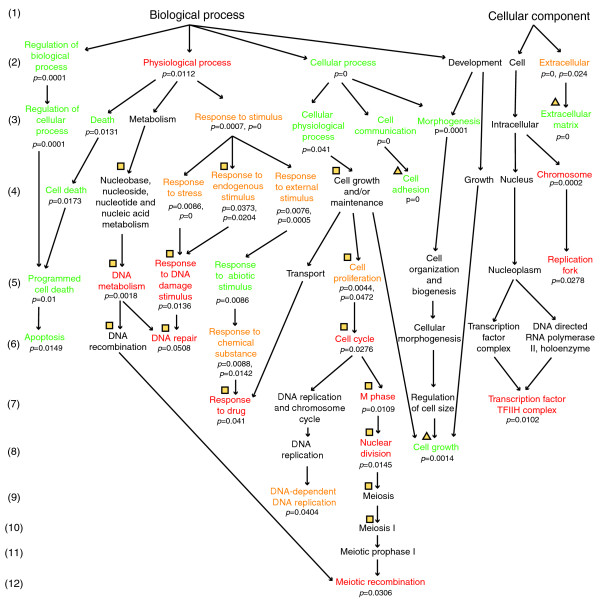
Directed acyclic graph representation of ontology terms. Association tree of the GO terms found significantly associated with *in vivo *innate drug responses. Bracketed numbers refer to the framework of the GO hierarchy and arrows indicate direct parent-child links. Relevant terms enriched in differentially expressed genes were computed using the GoMiner system [54]. Terms related to categories enriched in down-regulated genes, up-regulated genes or both down- and up-regulated genes are colored in green, red and orange, respectively, and the corresponding adjusted *p* values are indicated. Squares and triangles refer, respectively, to the terms relevant in the NODE547X and NODE519X gene modules.

To provide an overview of the enriched terms and their relationships within the framework of the GO hierarchy, an association tree was assembled for all selected genes. Primary and parent GO terms corresponding to the biological processes and cellular components were first retrieved. Then, the relevant terms were assembled in a directed acyclic graph representation, with arrows showing the parent/child relationships between terms and how all the selected terms relate to each other (Figure 5). This graph forms the basis on which an integrated representation of the molecular pathways involved in innate drug responses can be built.

### Systems view of molecular pathways underlying innate drug resistance

**Figure 6:**
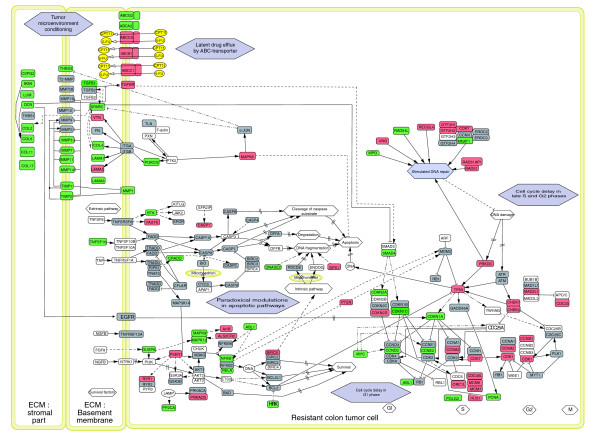
Systems view of a cellular state anticipating *in vivo *innate drug resistance. Molecular interaction networks characteristic of a resistant cellular state, prior to drug exposure, showing an integrated view of networks from resistant cells arrested either in G_1 _phase or in S-G_2 _phases. The representation recapitulates the dynamics and dependencies of gene modulations with specific components of the cell machinery identified through statistical comparisons, GO annotations and literature mining. Biochemical interactions are specified; the symbols used are derived from the standard nomenclature proposed by CellDesigner v2.5: arrow, direct activation; dotted arrow, unknown interaction; arrow with a strike through it, indirect activation; bar, inhibition; dotted bar, unknown inhibition; dashed arrow, transcriptional activation; dashed bar, transcriptional inhibition; arrow with clear arrowhead, transport. Red and green colors refer to up- and down-regulation, respectively, in resistant versus sensitive states; other genes analyzed with microarrays and/or Q-PCR are indicated in gray, and those not investigated in this study are indicated in white.

Information obtained through transcriptome analysis and complemented by literature and ontology-mining allowed identification of several molecular pathways that might be involved in the mechanisms underlying *in vivo *innate drug responses. The gene expression changes significantly associated with drug response states were mapped onto functional biological pathways using the CellDesigner v2.5 system [55], enabling representation of the dynamics and dependencies of each gene and gene module with specific components of the cellular machinery. The molecular interaction networks characteristic of the resistant state are shown in Figure 6, and stored using the Systems Biology Markup Language (SBML), a standard for representing biochemical networks [56], so that it will be possible in the future to use it as a starting point to model and simulate dynamic changes occurring during drug responses when appropriate software tools become available. At this stage, it provides a graphical overview of the major biological processes identified and of their relationships, which can serve as a guide to discuss the precise implications of the individual components based on how their expression is modulated in the resistant versus sensitive states prior to drug exposure.

### A cell cycle delay

Among the genes that govern cell cycle progression, *CHEK1 *and *CHEK2 *encoding the emergency response checkpoint proteins were highly expressed in the resistant state. CHEK1 is the damage-response kinase activated at early S-phase; CHEK2 is the replication-response kinase, which appears to be involved in regulating replication in response to perturbations at late S/G_2_, overlapping with CHEK1 functions. Such an emergency machinery may act to coordinate the cell cycle, slowing at the G_2_/M boundary, thus preventing the tumor cells from entering a mitotic catastrophe phase and reducing lethality due to premature mitosis [57]. At the onset of chemotherapy, those pre-activated checkpoint pathways might be able to operate rapidly by allowing inactivation of the CDC2-CyclinB (CDK1/CCNB1) complex, resulting in a G_2_/M arrest of the cell cycle. Interestingly, recent studies in cellular models reported chemical inhibitions of checkpoint kinases that might sensitize tumor cells to the induction of mitotic catastrophe, resulting in subsequent apoptosis [58,59]. Others proposed a role for the checkpoint kinases in early S-phase arrest, acting there by coordinating DNA synthesis with the cell cycle machinery through G_1_/S and G_2_/M checkpoints [60].

Additional control systems are composed of a family of protein kinases, the CDKs, which are in turn controlled by a series of proteins, including cyclin and cyclin-dependent kinase inhibitors (CKIs). The levels of expression of the genes encoding these proteins appear to be regulated in relation to the cell cycle in such a manner as to cause accumulation of cells either in mid-G_1 _phase as quiescent cells (G_0_-phase) or in the late S and G_2 _phases, both states characterizing innate resistance (Figure 6). First, the genes encoding major types of key regulators of CDKs appeared down-regulated, including *CCND2 *that encodes Cyclin D, which is active at mid-G_1 _phase to ensure G_1_-phase progression and is associated with CDK4/6, and *CCNE2 *that encodes Cyclin E, which appears later during G_1_-phase to sustain the cell cycle at the restriction point before entering S-phase (G_1_/S transition) and is abruptly destroyed upon entry into S-phase. Conversely, the genes encoding Cyclin A (*CCNA2*) and Cyclin B (*CCNB1*) appeared to be up-regulated (compare the Q-PCR analysis in Additional data file 9). The former associates with CDK2 (CDK2/CCNA2 complex) then with CDK1 (CDK1/CCNA2 complex) to drive cells through the S-phase and trigger the transition to G_2_; the latter is synthesized at the end of the S-phase and during G_2_, its activation being necessary for the transition from G_2 _to mitosis.

The genes encoding the cyclin-dependent protein kinases CDK1 and CDK7 were up-regulated, but no modulation of those encoding CDK2 and CDK4/6 was observed in the resistant state (compare the Q-PCR analysis in Additional data file 9). Moreover, expression levels of genes encoding three key CKIs believed to regulate CDK activities through G_1_-S-G_2 _transitions appeared to change in a cell cycle-dependent manner. *CDKN2D *(encoding p19, referred to as the INK4 inhibitor of CDK4 family) is highly expressed as expected during the S-phase, while *CDKN1A *(p21) and *CDKN1C *(p57, which preferentially bind to the G_1 _and G_1_/S class of CDKs) are down-regulated.

Consistent with these findings, numerous DNA replication functions appeared highly effective but with a slowed cell cycle S-phase in the resistant state compared to the chemo-sensitive one (Figure 6). For example, genes involved in initiation of DNA replication appeared up-regulated, such as those encoding the mini-chromosome maintenance proteins (*MCM6, MCM7*) and CDC45L, which were shown to interact and facilitate early steps of DNA replication at the level of the origin recognition complex (ORC1L; compare Q-PCR analysis in Additional data file 9). Down-regulated genes typically encode the proliferating cell nuclear antigen PCNA, which helps increase processivity of DNA polymerase. In addition, the *HUS1 *gene encoding a genotoxic stress-induced checkpoint complex component involved in chain elongation during DNA replication and response to DNA damage was highly expressed. Also, *CDKN1A *(p21) inhibition plays a positive regulatory role in S-phase DNA replication. This implies that previously reported specific inhibition of DNA replication occurring in anticipation of drug toxicity might not be essential for *in vivo *innate drug resistance. Conversely, slowing of the S-phase may occur through two indirect but related mechanisms, either by active repair of DNA damage and/or by increased mutagenesis, which may help cells to escape apoptosis [61].

Key players involved in DNA repair pathways appeared to undergo changes of gene expression in response to accumulation of cells in the S-G_2 _phases of the cell cycle (Figure 6). Most of these genes were significantly up-regulated in the resistant state, including those encoding the nucleotide excision repair (NER), the base excision repair (BER) and the double strand break repair (DSBR) complexes. Among them, three genes, *CDK7*, *GTF2H1 *and *GTF2H2*, encode components of the RNA polymerase II transcription initiation factor (TFIIH), which is involved in basal transcription and nucleotide excision repair. They were shown to play a direct role in transcription initiation and DNA repair. Uracil-DNA glycosylase (*UNG*) prevents mutagenesis by initiating the BER machinery [62]. Finally, some sub-pathways that play a role in DSBR appear to be modulated, such as the homologous recombination (HR) pathway that involves members of the RAD52 epistasis group, with up-regulation of *RAD51 *and *RAD51AP1*. Co-localization and interaction of the encoded proteins are essential for HR related to repair of DNA double-strand breaks (DSBs), facilitating DNA pairing and stimulating DNA recombination and repair [63]. *PRKDC*, which encodes the DNA-dependent protein kinase, a member of the non-homologous end joining (NHEJ) pathway, was up-regulated. *DNA-PK *encodes an activator of the XRCC4-ligase IV complex, which rejoins DNA ends [64]. Previous studies reported an active repair of drug-induced DNA damage associated with acquired drug resistance in mammalian cell lines or tumor samples [65,66]. The expression profile recorded in the resistant state suggests that *in vivo *stimulation of DNA repair pathways preexist drug exposure. It may be efficiently used at the beginning of chemotherapy and easily further amplified through programmed cell cycle arrest, resulting in a reduction of drug toxicity.

### Tumor microenvironment conditioning through deregulation of extracellular matrix turnover and remodeling

An integrated view of the recorded gene expression changes point to an unexpected role of ECM organization and its components in innate drug responses. The ECM is a dynamic network of intertwined macromolecules such as collagens, fibronectins, laminins and proteoglycans that exhibits structural and barrier functions, creating an influential environment for cellular dynamics [67]; a lack of renewal appears as one of the possible sources of innate drug resistance. In both tumors and metastases, a profound down-regulation of genes encoding both matrix proteins and modulators of matrix turnover was observed in the resistant state, compared to the steady expression levels characteristic of the chemo-sensitive state (Figure 6).

First, low levels of expression were recorded for the genes encoding the fibril-associated collagens type VI (*COL6A1, COL6A2*), the fibril-forming collagens type XI (*COL11A1*), a collagenous transmembrane protein type XVII (*COL17A1*) and the basement-membrane collagen type IV (*COL4A2*). Collagen type IV is the most important structural component of the basement membranes. Its network assembly is essential for the structural integrity and biological function of the basement membranes [68]. Collagen type VI is a major component of microfibrils in the extracellular space that has been reported to be needed for providing an appropriate extracellular environment [69,70]. These proteins interact with a broad range of molecules, including cell surface receptors such as integrins, basement membrane components such as collagen type IV, and proteoglycans, including biglycan (BGN) and decorin (DCN), two macromolecules shown to play a key role in storage deposits for growth factors and cytokines [71]. The expression of the genes encoding BGN and DCN, as well as those encoding a variety of glycoproteins, including laminins (*LAMA3, LAMB1*), and matricellular proteins such as SPARC and thrombospondins (*THBS3*), were down-regulated in the resistant state. The SPARC protein has been shown to actively contribute to the organization of the ECM. Its expression in basement membranes is usually restricted to tissues that undergo constant turnover and remodeling; a SPARC-null matrix was shown to be more easily degraded [72].

Conversely, no transcriptional modification was recorded for the genes encoding various integrins (I*TGA-1, -3, -4, -6, -7, -8 *and* ITGB-1-8*), a family of cell surface receptors that play a key role in integrating the ECM with the intracellular cytoskeleton. These results exclude the induction of integrins as a process to counterbalance a deficiency in the matrix of collagens. They are in contrast with previous studies in cellular models treated with the drugs that described either increased levels of ECM proteins associated with tumor drug resistance, such as laminin, collagen IV and collagen VI (reviewed in [73]), or apoptosis-induced drug resistance mediated through the binding of integrins [74].

A low expression of genes encoding matrix metalloproteinases (MMPs), a family of enzymes produced in the stroma compartment involved in the breakdown and remodeling of ECM proteins, was also observed in the resistant state. This is the case for collagenase-1 (*MMP-1*), stromelysin-1 and 3 (*MMP-3*, *MMP-11*), matrilysin (*MMP-7*) and membrane type T1-MMP (*MMP-14*) (compare Q-PCR analysis in Additional data file 9). However, no transcriptional modulation was recorded between the resistant and sensitive states for gelatinase-A and B (*MMP-2*, *MMP-9*), stromelysin-2 (*MMP-10*), epilysin (MMP-*28*), *MMP-19 *and *T2-MMP *(MMP-15) (α = 0.01). The tumor cells also exhibited down-regulation of genes encoding specific protease regulators such as TIMP-1 and TIMP-2. Both protease inhibitors were shown to act in the regulation of the proteolytic activity of stromal MMPs, the balance of ECM-degrading proteinases and their inhibitors being a crucial parameter to promote degradation, and are well known inhibitors of angiogenesis [67]. In addition, TIMP-1 and TIMP-2, in concert with MT1-MMP, were found to bind to and activate proMMP-2 and proMMP-9, respectively [75]. A deficit in the concentrations of both TIMPs and MT1-MMP may be deleterious for both activation of MMP-2 and MMP-9, considering that the expression of the latter remained unchanged.

Previous studies of cell adhesion molecules (CAMs), such as CD44 and E-cadherin (CDH1), cell surface glycoproteins that are important in cell-cell and cell-matrix interactions, cell growth and differentiation, reported that their expression was significantly associated with tumor grades and depth of primary tumor invasion [76]. Consistent with those findings, increased expression of the *CD44 *gene and decreased expression of the *CDH1 *gene were observed in tumoral versus normal/non-tumoral colons, confirming high tumor grade and increased primary tumor invasion, while no transcriptional modulation was observed for those genes between the resistant versus sensitive groups of samples (α = 0.01). Thus, decreasing concentrations of active proteases (MMPs) and proteases inhibitors (TIMPs), and limiting concentrations of structural collagens, proteoglycans and glycoproteins show direct relationships with primary responses associated with drug resistance compared to the steady expression levels found in the chemo-sensitive state. The temporal and spatial regulation of ECM turnover and reassembling events appear deficient in the resistant state, while they are required for adaptation to constantly changing environments and active cellular responses to external stimuli. This results most likely in a static ECM organization, a low matrix-degrading capacity that limits ECM reloading and matrix micro-environment remodeling, a decreased activity of ECM-producing cells and a limitation of downstream activities of ECM components [77].

Tumor cells appear to play a role in conditioning the neighboring stroma/basement membrane to induce a protective micro-environment. In the recorded expression profiles, the differences observed between the chemo-sensitive versus resistant states were not attributable to varying amounts of stroma versus tumor epithelium among patient samples as no transcriptional modulation of the genes encoding markers characteristic of epithelial tumor cells (cytokeratins, *KRT4, 5, 7, 8, 13, 14, 15, 17, 18, 19*) and of stromal cells (vimentin, *VIM*) could be measured (α = 0.01). These observations are consistent with results reported in other studies indicating a relatively small contribution of stromal cell to tumor expression profiles in macro-dissected samples similar to those used in the present study, while micro-dissection appeared to introduce significant experimental biases due to important differences occurring during manipulation of very small samples [78,79].

A static organization of the ECM components might contribute to innate drug resistance of a solid tumor by preventing penetration of therapeutic agents, but it might also protect cells from chemotherapy-induced deleterious effects by disrupting specific signaling connections. As proteases and their inhibitors were shown to target many other non-ECM proteins, including growth factor receptors, cell-associated receptors and cytokines [80], the observed imbalance of their activities in the resistant state might also have a negative regulatory effect on physiological mechanisms such as angiogenesis and cell apoptosis that are associated with drug sensitivity [67,80].

### Paradoxical modulations of apoptotic pathways

Although apoptosis was reported as one of the most obvious targets for cancer treatment because chemo-sensitivity requires an intact cellular signaling machinery that participates in programmed cell death [81-83], only puzzling gene modulations were observed prior to drug exposure. Out of 40 genes associated with one aspect of apoptosis or another, 17 were over-expressed and 23 under-expressed, encoding several pro- and some anti-apoptotic proteins. Many of the expression differences seemed to occur in antagonistic directions, probably reflecting disorganized or simply heterogeneous apoptotic machinery responses and are not discussed in detail here (Figure 6). This suggests that apoptosis inhibition observed in drug resistant cell lines may reflect the singularity of the corresponding models, and is not likely to occur in solid tumors prior to drug exposure. The nuclear factor kappa B (NF-kB) gene *NFKB1 *[84] was mainly under-expressed in the innate resistant state. This observation contrasts with a previous report in which NF-kB inhibitors were proposed to circumvent drug resistances [85]. It is, however, in agreement with another study that reported a two-stage 'permissive apoptosis-resistance' mechanism based on analysis of a human prostate cancer cellular model [37]. The same mechanism is apparently not involved in the innate resistant state, as down-regulation of the NF-kB-stimulated genes or inhibition of the TGF-β pathway, which could be expected if that was the case, were not observed.

### Latent drug efflux by ABC-transporters

Finally, it appears that the high expression levels of genes encoding ABC-transporters recorded in the resistant versus sensitive states may play a role in innate drug responses. This may empower the cells with the ability to pump out drugs even before they are exposed to them, thus decreasing drug intake at the onset of chemotherapy. Active efflux of xenobiotics is a key mechanism of cell adaptation to environmental stress. Namely, three ABC-transporter genes, *P-gp *(*ABCB1*), *MRP1 *(*ABCC1*) and *MOAT-C *(*ABCC5*), were up-regulated in the resistant state (Figure 6), compared to their low expression levels measured in the chemo-sensitive state. The ABCC1 and ABCC5 proteins, members of the MRP subfamily, function as a multi-specific organic anion transporter or contribute to the degradation of phosphodiesterase, respectively. ABCB1, a member of MDR/TAP subfamily, encodes an ATP-dependent drug efflux pump for xenobiotic compounds with broad substrate specificity. Both ABCB1 and ABCC1 have been shown to be responsible for decreased drug accumulation in multidrug resistant cells [86,87]. By contrast, down-regulation was observed in the resistant state for *ABCA2*, which encodes a member of the ABC1 subfamily, or *ABCG2*, which encodes a member of the MDR/TAP subfamily (compare Q-PCR analysis in Additional data file 9). Interestingly, this last result contradicts a previous study [30] but is in agreement with another [32]. This apparent discrepancy most likely reflects the fact that the former study reported data obtained with cell lines and tumor samples from patients under the influence of the drugs, while the latter focused on tumor samples prior to drug exposure. This highlights some predictable differences between drug-induced modulations of transporter gene expression and primary innate drug responses.

## Conclusions

Clinical drug resistance is a major concern in the treatment of human cancers, most of them being resistant to therapy at the time of drug presentation. Deciphering the molecular mechanisms that contribute to such innate drug resistance should improve both the prediction of treatment failure and the development of new strategies to overcome resistance. Our study reports for the first time a systems approach toward understanding the *in vivo *cellular states of clinical samples collected from CRC patients prior to their exposure to a combined chemotherapy.

As shown here, microarray technology has the potential to complement classic biological and clinical parameters, with important implications for the assessment of complexity, redundancy and interdependence in biological pathways involved in drug responses. The ability to automate the assessment of changes in genome-wide gene expression, combined with vigilant experimental design and statistical power simulations to optimize use of relevant biological samples, facilitates their evaluation for clinical significance. Through transcriptome analysis and functional annotation, we were able to highlight functional molecular interaction networks that may contribute to primary drug responses. The results obtained suggest that the innate resistant state may be characterized by: poorly dividing tumor cells as deduced from observed accumulation of cells in mid-G_1 _phase and decreased DNA replication processivity; an increased DNA repair associated with cell cycle delay in late S and G_2 _phases preventing occurrence of mitotic catastrophe and cell apoptosis [58,59]; an increased drug efflux potential by ABC-transporters pre-existing drug exposure; and a dysfunctional ECM with decreased renewal ability of ECM and basement membrane components, most likely resulting in decreased stimulation of angiogenesis.

The multiplicity of molecular mechanisms that appear to be involved in the innate resistant state emphasizes the difficulty of influencing cell responses to drugs. This is illustrated by the limited success of recent therapeutic attempts focused on individual targets such as CHECK, MMP, SPARC or P-gp [75,87-90]. Hence, the functional interaction molecular map proposed herein, as it provides a more global systemic view of cellular states, constitutes a helpful starting point to identify by-pass chemotherapy schemes designed to stimulate or inhibit a variety of targets simultaneously with the potential of enhanced treatment effectiveness.

The information generated in this study might also provide new potential markers for prediction of the chemo-sensitive and resistant states to the combined chemotherapy in newly diagnosed cancer patients, allowing therapeutic adjustment. Because of the limited size of our study, the genes identified as differentially expressed require further evaluation in a larger patient cohort using either microarrays for additional exploration or quantitative RT-PCR for fast, low-cost assessment. Further integration with data collected at the genomic level through mutation analysis, at the level of the entire transcriptome by complementary comprehensive methods such as massive parallel signature sequencing [91], at the proteome level with emerging global technologies such as isotope-coded affinity tags coupled with mass spectrometry [92], and at the metabolome level by mass spectrometry or NMR [93] should provide the basis for designing reliable predictive markers and deciphering the molecular pathways involved in drug responses.

The research strategy developed in this study represents a required and valuable step for establishing an initial systems view of molecular pathways underlying innate drug resistance and should be also applicable to other physiological and pathological situations.

## Materials and methods

### Tumor specimens and protocol

Forty tumor biopsies, including left-sided colon tumors (T, *n* = 12), adjacent non-tumoral colons taken near the tumor (N, *n* = 12) and liver metastases (M, *n* = 16), were obtained from fifteen patients enrolled at the Centre Régional de Lutte contre le Cancer (CRLC, Montpellier, France) during a two-year period in a FOLFIRI phase II clinical trial [94]. The diagnosis was determined by pathological examination using the following criteria: sporadic advanced metastatic colorectal cancer; histological verification of disease; patients between the ages of 18 and 75 years; World Health Organization (WHO) performance status of 2 or less; no history of prior chemotherapy or any treatment with TOP1 drug inhibitors; and tumor markers (ACE, CA19.9, LDH) evaluation at baseline [94]. Written informed consent was obtained from the patients prior to enrollment for collection of the samples for medical research under a protocol approved by the local ethical committee (CCPRB COD 03). Sections of patient biopsies (>3 mm^3^) were reviewed by a pathologist prior to analysis, including localization, measurement of the tumor and assessment of margins. Clinical data such as diagnosis, site, stage, age and sex are summarized in Additional data file 10. Patient biopsies were snap frozen with liquid nitrogen prior to RNA extraction and stored at -196°C.

After surgery, all patients received every two weeks comparable regimens of first-line chemotherapy, corresponding to a simplified 5-FU/FA regimen combined with CPT-11 at 180 mg/m^2 ^at C1, increased to 220 mg/m^2 ^at C2 and to 260 mg/m^2 ^at C3 and subsequent cycles if the toxicity grade remained less than 3 [94]. Initial (primary) response rates were assessed after each series of two treatment cycles based on WHO response criteria [8], considering complete or partial regression, stabilization or progression of the disease based on the evaluation of growth and records of diagnosed liver metastases, as described in [11].

### Microarray design and manufacture

The human cDNA microarrays used contained 11,520 sequences derived from various sequence-verified clone collections, including a Mammalian Gene Collection human sequence-verified subset (9,600) [95], a private collection of 1,536 human clones identified by a unique IMAGE Clone ID [96] and 384 control calibrators (Lucidea™ Universal Scorecard™, Lucidea™ microarray Scorecard™ v1.1, Amersham Biosciences, part of GE Healthcare, Palo Alto, CA, USA; and SpotReport™-3 array validation system, Stratagene, La Jolla, CA, USA). The array set provides a genome-wide coverage of functional pathways, such as cell cycle and checkpoints, cell growth and/or maintenance, cell adhesion and proliferation, development, extracellular matrix, apoptosis, response to DNA damage and DNA repair, DNA replication, transcription and RNA processing. High confidence qualifications and annotations of the clone collections used the Genexpress workbench as described in Additional data file 2. The corresponding data are available through our web site [97].

All arrays were printed in the laboratory on amino-modified mirrored glass slides using the Lucidea™ array spotter (Amersham Biosciences) as described in Additional data file 2. The suite of amplified cDNAs was printed as a group in two spatially separated replicates.

### Nucleic acid extraction

Frozen sections from each tumor biopsy were examined microscopically prior to RNA extraction to confirm that the sample was representative and contained more than 50% tumor cells [30]. Total RNA was extracted using RNeasy columns (Qiagen Ltd, Hilden, Germany) according to the manufacturer's instructions. RNA purity and quantity was assessed by UV measurement. RNA integrity was judged using RNA 6000 nano chips and the Agilent 2100 Bioanalyzer (Agilent Technologies, Palo Alto, CA, USA) according to the manufacturer's instructions. RNA quality-control was performed using user-independent classifiers as described in [98].

### Hybridization experimental design

Microarray analyses were applied to tumor and metastasis biopsies from 13 individuals following a randomized and blinded unbalanced design. Additional human normal colon samples (C_A_, C_B_, C_C_), one colon adenocarcinoma sample (C_T_) and one normal liver (C_L_) tissue sample (Stratagene) were included as calibration controls but were not considered in statistical analyses. Uneven numbers of samples were randomly allocated to each of the engineers who were not aware of sample phenotypes. To assess data reproducibility and minimize dye bias effects, each of the samples was measured four times, twice with Cy3 and twice with Cy5. To ensure robustness and flexibility in data analysis, a reference design was used with a universal reference sample (Stratagene) serving as a baseline for the comparisons of tumor samples. Such a design does not require pre-definition of the subgroups for comparison, allows robust discovery of non-anticipated classes among the samples and is compatible with subsequent additional sampling.

Statistical power (1-β) for *t* statistics of the experimental design was computed for estimation of false negatives (FNR) and FDRs [99] as shown in Additional data file 3. This calculation is based on the observation that the gene-specific expression measurements are approximately normally distributed, and takes into account the accepted confidence level (α), the magnitude of the effect measured (Φ), the biological variation (σ) expected in the population investigated, and the size of the groups of samples from individual patients (n1, n2). Statistical comparison was done considering that biopsies collected prior to drug exposure may be subsequently categorized in two groups of chemo-sensitive (complete and partial responses) or resistant (progressive and stable diseases; Additional data file 10) samples in view of the initial response rates of individual patients to combined chemotherapy [11].

### Gene expression profiles

RNA for hybridization was prepared by amplification using a modified Eberwine protocol [100]. Reverse transcription, *in vitro *transcription and amplification factors were used for amplified-RNA quality control (Additional data file 2). One microgram of each amplified RNA and human universal reference RNA (Stratagene) were labeled alternatively with Cy-5-dCTP and Cy-3-dCTP (Amersham Biosciences). Following a vigilant quality control procedure based on three levels of qualification (RNA quality, linear amplification and target synthesis specifications) using a set of 16 specific parameters (described in Additional data file 2; see also [51,98]), 26 among the 40 samples were included in the microarray workflow (Additional data file 10). Hybridizations to the arrays were performed as described in Additional data file 2; array images and raw data were obtained using the GenIII array scanner (Amersham Biosciences) and ArrayVision™ 7.0 software (Imaging Research Inc., Amersham Biosciences, Palo Alto, CA, USA). Raw data were first imported into a Genetraffic™ duo database (Iobion Informatics, Toronto, Canada), local background-subtracted and normalized using a Lowess (locally weighted linear regression) transformation [101]. The following selection criteria were applied: all spots having a mean signal (after background subtraction) less than that of the background and below that of the negative controls in both Cy3 and Cy5 channels were systematically excluded; the data were also filtered to exclude spots flagged as missing or corrupted in one array. We next calculated the average expression ratios (test/reference) in all analyses. Log_2 _values of lowess-transformed data were used for all subsequent statistical analyses. For reporting genes by name, IMAGE Clone IDs corresponding to the microarray probe sequences were used to extract UniGene Cluster IDs and names (Build 184, June 2005). For genes represented by multiple probes (that is, different clones corresponding to the same gene) on the array, each probe and the related expression ratios were considered and reported separately. MIAME-compliant data [102] have been deposited in Gene Expression Omnibus (GEO) at NCBI [103] and are accessible through GEO Series accession number GSE3964.

### Quantitative RT-PCR analysis

The expression of selected genes was also analyzed by Q-PCR using the TaqMan^® ^low density Micro Fluidic Card or TaqMan^® ^single assays (Applied Biosystems, Foster City, CA, USA), according to the manufacturer's instructions.

Total RNA (1 μg) from tumor biopsies from 15 individuals prepared as described above was used in the Q-PCR reactions (Additional data file 10). cDNA was prepared using the high-capacity cDNA archive kit (Applied Biosystems) as described in the manufacturer's instructions. Preliminary experiments using 18S rRNA TaqMan^® ^probes were carried to test the efficiency and reproducibility of the reverse transcription. The expression levels were determined using the Applied Biosystems 7900-HT SDS instrument; duplicate threshold cycle (Ct) values were averaged, and transformed using a relative quantification method [104]. Fold changes for each tested gene (target gene) was normalized to the *GUSB *housekeeping gene (reference gene) and compared to the relative median expression of all samples (calibrator sample), using the formula Fold change = 2^-ΔΔCt^, where ΔΔCt = (C_t-target _- C_t-reference_)_Sample-n _- (C_t-target _- C_t-reference_)_Calibrator sample_. Sample-n corresponds to any sample for the target gene normalized to the reference gene and the calibrator sample represents the 1 × expression of the target gene normalized to the reference gene considering all tested samples.

The data are expressed as mean 2^-ΔΔCt ^of each group of samples (resistant and sensitive states) and lower and upper bound mean of 95% confidence intervals. Differences between groups were examined for statistical significance using *z*-statistics, with FDR *p* values adjustment (α = 0.05). Expression changes in the same direction in both microarray analysis and Q-PCR, and *p *< 0.05 was considered statistically significant.

### Statistical analysis of cDNA microarray data

Centered Pearson correlation was used as a similarity measure to visualize the expression patterns among samples. Average linkage hierarchical clustering analysis was implemented using the Cluster program and the results were displayed using TreeView [105]. The distances between samples are represented on a dendrogram as shown in Figure 1.

*t* statistics with permutation-based *p* values adjustment were used to select clusters (nodes) of genes significantly differentially expressed between two groups using the Cluster Identification Tool [106]. The group labels (resistant and sensitive states) were randomly permuted and the *t* statistic for each gene node in the permuted data set was calculated. The process was repeated 10,000 times. A *p* value was reported for each gene node by comparing the observed statistic with the permutation statistics. Only gene nodes with *p* values < 0.05 were considered relevant.

ArrayStat 1.0 software (Imaging Research Inc.) was used for parametric analysis. For the data, only elements for which at least one-third of the measurements across all samples had values were included. A pooled curve-fit error method was used for random error estimation; a range of 3.0 median absolute deviations (MADs) to 4.0 MAD established outlier-detection thresholds automatically. Then, groups were compared as independent conditions by a *z*-test (α = 0.01). In this step, data were subjected to iterative normalization by centering to the median across groups. For multiple testing corrections, the FDR procedure was used [107]. Only genes whose expression significantly differed between the resistant and sensitive states (*p* values < 0.001) were selected.

### Bioinformatics

Gene name, aliases, representative accession number, Locuslink IDs (LLID), UniGene Cluster IDs (Hs.) and GO IDs [52], chromosome and sub-cellular localization of genes from microarrays were obtained from the SOURCE browser [108,109]. Attributes were collected using the individual IMAGE Clone ID, corresponding to the microarray probe sequences. A complete GO annotation and descriptions [52] were obtained from QuickGO, a browser for the GO data at the European Bioinformatics Institute [110].

### Ontology enrichment

Genes had previously been categorized into various ontologies and pathways. If a particular ontology term was enriched for genes that are significantly expressed in response to the process under study, it was concluded that the ontology term is likely to be involved in the process. EASE online [53,111] via the LLID and GoMiner [54,112] via the gene symbol were used to analyze the lists of up- and down-regulated genes for GO categories [52] that were significantly statistically over-represented. EASE scores were used as upper bound of the one-sided jackknife Fisher's exact probability test and GoMiner that estimates the *p* value using two-sided Fisher's exact test of *p* values. Only GO categories that had *p* values of less than 0.05 were reported.

### Networks searches

Differentially expressed genes were mapped onto relevant biological themes and represented using the CellDesigner version 2.5 program [55], a structured diagram editor that represent the dynamics and dependencies of gene modulations with specific components of the cellular machinery. Gene expression data were displayed using the HUGO symbol nomenclature by color-coding over-regulated genes in red, down-regulated genes in green, genes present onto the microarray in gray and untested genes in white. Networks are stored using SBML, a standard for representing biochemical networks [56].

## Additional data files

The following additional data are available with the online version of this paper and can also be obtained through our web site [113]. Additional data file 1 outlines the workflow of the experimental design and analysis strategies. Additional data file 2 is a comprehensive description of the experimental procedures used in this study. Additional data file 3 is a spreadsheet file including the statistical power simulations. Additional data file 4 is a spreadsheet file including descriptive statistics and annotation of differentially expressed genes. Additional data file 5 is the gene expression matrix of the significant node NODE519X. Additional data file 6 is the gene expression matrix of the significant node NODE547X. Additional data file 7 is a serial representation of the relative expression of a series of genes collected by Q-PCR during the validation step performed on a new metastasis sample collected from an additional patient. Additional data file 8 is a spreadsheet file including the search results for significantly enriched GO classes. Additional data file 9 is a spreadsheet file including Q-PCR analysis collected for additional genes representative of key biological processes. Additional data file 10 is a spreadsheet file that contains the information relative to the patients and tumor samples used in this study, and QC selections [52,95,98,100,114-122].

## Supplementary Material

Additional data file 1 The workflow of the experimental design and analysis strategies. Click here for file

Additional data file 2 A comprehensive description of the experimental procedures used in this study. Click here for file

Additional data file 3 A spreadsheet file including the statistical powersimulations. Click here for file

Additional data file 4 A spreadsheet file including descriptive statistics and annotation of differentially expressed genes. Click here for file

Additional data file 5 The gene expression matrix of the significant node NODE519X. Click here for file

Additional data file 6 The gene expression matrix of the significant node NODE547X. Click here for file

Additional data file 7 A serial representation of the relative expression of a series of genes collected by Q-PCR during the validation step performed on a new metastasis sample collected from an additional patient. Click here for file

Additional data file 8 A spreadsheet file including the search results for significantly enriched GO classes. Click here for file

Additional data file 9 A spreadsheet file including Q-PCR analysis collected for additional genes representative of key biological processes. Click here for file

Additional data file 10 A spreadsheet file that contains the information relative to the patients and tumor samples used in this study, and QC selections. Click here for file

## Figures and Tables

**Table 1:** Selection of 30 genes considered for further Q-PCR analysis

	Symbol	Clone ID	M	T	T&M	All tumor samples (T&M)			
						
						Intensity	Ratio	Min-value	Max *P* value
1	ABCA2	3508171	0	0	1	411	-1.4	4.36E-05	4.93E-05
2	ABL1	345666	1	0	0	569	-1.5	1.44E-04	1.44E-04
3	CASP1	486506	0	1	0	714	1.9	6.30E-06	6.93E-06
4	CHEK1	3530606	1	0	1	1,901	1.6	1.66E-05	3.89E-05
5	CHEK2	3532866	0	0	1	2,624	1.4	2.72E-05	3.77E-05
6	COL4A2	3507072	0	0	1	5,813	-1.6	7.86E-03	7.86E-03
7	COL6A1	3506644	1	1	1	4,268	-2.6	4.43E-12	3.63E-02
8	COL6A2	3347413	1	0	1	3,906	-4.3	1.37E-11	1.31E-02
9	FOS	3688670	1	0	1	8,312	-1.7	7.39E-05	1.28E-04
10	HRASLS3	3051149	0	1	0	1,814	-1.7	6.22E-05	6.22E-05
11	LAMB1	428443	1	0	1	2,747	-1.5	5.06E-06	2.93E-04
12	MAD2L1	2964388	1	0	1	4,141	1.6	2.94E-05	1.99E-04
12	MMP11	725180	1	0	1	2,917	-2.3	1.78E-05	3.42E-02
14	PEA15	3346270	0	0	1	1,366	-1.3	3.48E-04	3.48E-04
15	RAD51	3139011	1	0	0	1,630	1.9	9.78E-03	9.78E-03
16	RECQL4	3634266	1	0	1	3,372	1.6	9.13E-06	1.76E-05
17	RERG	3357341	1	1	1	294	-3.6	1.17E-05	3.63E-02
18	RRAGD	3536088	1	0	1	596	-2.6	6.70E-07	4.35E-02
19	SERPINF1	2961120	1	0	1	1,902	-1.5	8.33E-10	2.74E-05
20	TGFBI	2958878	1	1	1	13,900	-2.4	5.43E-10	1.31E-02
21	TOP2B	470615	1	0	1	495	1.5	6.15E-06	2.34E-04
22	TP53	3544714	1	0	1	4,178	1.8	1.06E-06	1.32E-05
23	*BCL2*	*232714*	*0*	*0*	*0*	787		Not significant	
24	*CES2*	*4298532*	*0*	*0*	*0*	6,538		Not significant	
25	*E2F4*	*3504728*	*0*	*0*	*0*	5,620		Not significant	
26	*ITGB6*	*759142*	*0*	*0*	*0*	648		Not significant	
27	*JUN*	*3606344*	*0*	*0*	*0*	2,037		Not significant	
28	*RAD52*	*3614681*	*0*	*0*	*0*	322		Not significant	
29	*TGFB1*	*3356605*	*0*	*0*	*0*	2,188		Not significant	
30	*TOP1*	*502960*	*0*	*0*	*0*	3,484		Not significant	

For each gene, symbol and Clone ID are indicated. M, T and T&M columns (1 = present, 0 = absent) specify, in the respective group of samples, which genes were statistically differentially expressed using microarrays (α = 0.01); italics refers to genes for which differential expression was 'not significant'. The relative expression levels recorded with microarrays are indicated as the ratio between resistant and sensitive samples, specified as negative (down-regulated) or positive (up-regulated) values, together with the intensity measured in resistant colon tumors. Adjusted *p* values were computed using *z* statistics with false discovery rate corrections, α = 0.01; columns 'Min *p* value' and 'Max *p* value' refer to lower and upper bound *p* values, respectively.

**Table 2:** Validation of microarray gene expression data

Symbol	Assay ID	Tumors				Metastases			
		
		Ct_R_	Ct_S_	Ratio	*p* value	Ct_R_	Ct_S_	Ratio	*p* value
**Fully validated genes**									
CHEK1*	CHEK1-Hs00176236_m1	30.3	30.9	2.2	2.11E-02	29.8	31.9	4.0	2.87E-03
CHEK2*	CHEK2-Hs00200485_m1	29.3	30.1	2.5	3.40E-04	29.0	30.9	2.0	8.33E-03
**COL4A2**	COL4A2-Hs00300500_m1	25.2	23.5	-3.7	1.60E-06	24.9	23.5	-4.3	5.27E-07
**COL6A1**	COL6A1-Hs00242448_m1	24.8	21.9	-6.6	3.45E-08	25.9	24.6	-2.0	9.54E-03
**COL6A2**	COL6A2-Hs00365167_m1	24.8	21.6	-8.6	8.66E-08	25.1	24.0	-2.8	5.40E-05
**FOS**	FOS-Hs00170630_m1	21.5	21.1	-2.7	2.36E-03	22.1	21.6	-3.0	2.86E-06
**HRASLS3**	HRASLS3-Hs00272992_m1	27.6	25.2	-5.6	3.42E-07	25.7	25.7	-2.4	2.14E-05
**LAMB1**	LAMB1-Hs00158620_m1	24.6	23.4	-2.4	5.25E-04	25.3	24.1	-2.8	8.46E-06
**MMP11**	MMP11-Hs00171829_m1	25.4	23.6	-3.1	1.85E-05	25.6	23.0	-10.2	2.46E-08
**PEA15**	PEA15-Hs00269428_m1	24.4	23.5	-2.4	4.52E-04	24.5	23.7	-2.3	2.50E-05
**RAD51**	RAD51-Hs00153418_m1	26.6	27.7	2.3	9.81E-03	26.2	28.0	3.0	4.23E-03
RECQL4*	RECQL4-Hs00171627_m1	27.6	28.3	2.1	1.26E-03	27.0	29.0	3.7	2.41E-03
**RERG**	RERG-Hs00262869_m1	31.8	28.5	-11.1	5.07E-12	31.2	30.5	-3.4	2.10E-06
**RRAGD**	RRAGD-Hs00222001_m1	29.6	27.0	-7.8	1.74E-07	28.5	26.9	-3.0	2.60E-05
**SERPINF1**	SERPINF1-Hs00171467_m1	26.1	23.7	-3.5	2.52E-05	24.6	24.7	n/a	n/a
**TGFBI**	TGFBI-Hs00165908_m1	23.9	22.0	-5.4	2.35E-06	24.0	21.6	-6.7	7.27E-08
**TP53**	TP53-Hs00153340_m1	24.0	25.2	2.5	7.00E-03	24.0	25.4	2.9	3.21E-03
*BCL2*	*BCL2-Hs00608023_m1*	29.7	29.5	n/a	n/a	29.2	29.5	1.1	6.86E-01
*E2F4*	*E2F4-Hs00608098_m1*	23.0	22.6	-1.1	8.34E-01	22.8	23.2	-1.3	1.94E-01
*ITGB6*	*ITGB6-Hs00168458_m1*	27.5	27.8	-1.0	9.16E-01	26.9	27.7	-1.0	9.32E-01
*RAD52*	*RAD52-Hs00172536_m1*	29.4	28.5	-1.9	1.12E-02	28.5	28.6	-1.1	5.42E-01
*TOP1*	*TOP1-Hs00243257_m1*	25.1	24.8	-1.1	7.89E-01	24.6	26.2	1.1	8.14E-01
**Validated genes**									
**CASP1**	CASP1-Hs00354832_m1	26.6	27.6	2.1	5.91E-03	28.6	28.3	-1.5	1.80E-01
**MAD2L1**	MAD2L1-Hs00829154_g1	26.7	27.5	2.0	9.91E-03	27.0	27.7	1.4	2.72E-01
**False discoveries**									
ABCA2	ABCA2-Hs00242232_m1	26.2	26.0	1.0	9.94E-01	25.9	26.0	-1.1	6.59E-01
ABL1*	ABL1-Hs00245445_m1	26.2	25.7	-1.3	3.05E-01	26.2	26.1	-1.3	2.91E-01
TOP2B	TOP2B-Hs00172259_m1	25.4	25.1	-1.2	3.19E-01	25.1	25.6	-1.1	5.46E-01
*CES2*	*CES2-Hs00187279_m1*	26.2	24.6	-2.8	5.41E-04	24.2	24.6	2.0	2.33E-02
*JUN*	*JUN-Hs00277190_s1*	22.8	22.1	-1.6	1.86E-02	22.1	22.3	-2.5	9.00E-06
*TGFB1*	*TGFB1-Hs00171257_m1*	25.8	23.8	-3.5	2.70E-06	24.8	24.5	-2.2	5.83E-05

Q-PCR validation step on a series of 30 genes. For each gene, symbol and TaqMan^® ^assay (assay ID) are indicated; asterisks refer to TaqMan^® ^single assays. Genes displayed in bold were considered for further Q-PCR analysis of new samples from additional patients (compare Figure 3 and Additional data file 7); italics refer to genes selected as not significantly differentially expressed with microarrays. The threshold cycle Ct_S _refer to chemo-sensitive states and Ct_R _to resistant states; the expression changes (ratio) are specified as negative (down-regulated) or positive (up-regulated) values between resistant versus sensitive samples. Adjusted *p* values were computed using *z* statistics with false discovery rate corrections (α = 0.05). n/a, not analyzed.
