# Supplementary material for: Deciphering cellular states of innate tumor drug responses
Source: Genome Biol. 2006 Mar 15;7(3):R19. doi: 10.1186/gb-2006-7-3-r19 (PMC1557757; doi:10.1186/gb-2006-7-3-r19)
Supplement: Additional data file 1 — The workflow of the experimental design and analysis strategies. [file gb-2006-7-3-r19-S1.doc]

| **Experiment** | **Data** | **Analysis** | **Exploitation** |
| --- | --- | --- | --- |
| Patient  1) Surgery  N T M  RNA  cDNA array  Hybridization  Normalization  Filtering  2) Subsequent  chemotherapy  adjustment  of treatment  Functional  validation | Sample size  n genes  Sample  heterogeneity  (CV)  Observed  group size  Expected  group size  Drug response phenotype  **resistant**  **sensitive** | Statistical  power  Hierarchical  clustering  Statistical analysis  Selection of  discriminant genes  **Prognosis of**  **drug response** | Cancer-related  Genes  Other genes  Annotation  Enrichment in  GO terms  Molecular  pathways  **Working hypotheses on mechanism of drug responses** |

**Additional Data File 1: Microarray experimental workflow and data analysis strategy**

Workflow on the key steps of Experimental design (Experiment), Data structure (Data), Data management (Analysis) and Knowledge extraction (Exploitation). The combination of shapes (e.g., circles, squares, ovals, etc) and arrows are basic representations of processes and flow (control and data) as found in data flow diagrams and flow charts. The symbols N, T and M refer to non tumoral colons, primary colon tumors and liver metastases, respectively.
