# Supplementary material for: Deciphering cellular states of innate tumor drug responses
Source: Genome Biol. 2006 Mar 15;7(3):R19. doi: 10.1186/gb-2006-7-3-r19 (PMC1557757; doi:10.1186/gb-2006-7-3-r19)
Supplement: Additional data file 2 — A comprehensive description of the experimental procedures used in this study. [file gb-2006-7-3-r19-S2.doc]

**GRAUDENS ET *AL.*: ADDITIONAL DATA FILE 2**

**- EXPERIMENTAL PROCEDURES ONLINE -**

**- Complementary DNA array design and manufacture**

Human cDNA Clone collection

Clone Processing

Array printing

**- Gene expression measurements**

RNA purification

T7-linear amplification

Targets labeling

Hybridization

**- Quality assessment workflow**

Quality Controls of RNA samples

Quality Controls of IVT transcription

Quality Controls of Cy-Dye targets labeling

Quality Controls of PCR probes synthesis

**- Statistical precision and power simulations**

**Complementary DNA array design and manufacture**

***Human cDNA Clone collection.*** An assembled sequence-verified collection of 11,136 human cDNA clones was selected from the I.M.A.G.E. Consortium resources (<http://image.llnl.gov/>). A total of 9,600 clones were derived from the NCI_MGC_IRAL libraries of the Mammalian Gene collection [95], and complemented with 1,536 cancer-targeted clones. These clones are sequence-verified and many of them even fully sequenced. Quality control measures of sequence information were implemented in order to ensure that the clone collection is accurate. All clones were checked for identity and absence of contaminating microorganisms.

Supplemental computation was performed to permit gene-centric analyses of the clones collection, and place each clone into its biological context, using the Genexpress workbench for automatic sequence and annotation viewing and editing, as described in [114]. Precisely, using a series of sequence analysis and information integration tools, significant data were extracted from various public databases, including Unigene [115], TIGR [4, 5], H-INV [118], SWISSPROT [119], e-VOC [120], GO [52], LLID [115], and Genbank [121]. The information collected was curated, integrated, and the results displayed in such a way that the various annotations can be compared.

***Clone Processing*.** Frozen glycerol stocks of bacteria containing individual cDNA clones, distributed through the I.M.A.G.E. Consortium in 384-well plate format, were purchased from the RZPD resource center (Germany). The bacterial clones were inoculated into 96-well flat bottom blocks containing LB/chloramphenicol (12.5-25 µg/ml) or LB/ampicilline (25 mg/ml) and incubated overnight at 37°C with agitation. Plasmid DNAs were prepared using robotics stations (QIAprep 96 turbo BioRobot kit; BioRobot 8000, Qiagen, Germany). PCR amplification of DNA with primers specific for the vector sequences flanking the cDNA insert was performed in 96-well PCR plates in a Perkin-Elmer 9600 thermocycler (Perkin Elmer, USA) in 50 µl reactions containing 1x detergent-free PCR buffer (Finnzyme, USA), 0.2 mM dNTPs, 0.4 µM of each primer, 1 unit of DyNazyme™ EXT DNA Polymerase (Finzymes, USA) and 1 µl of plasmid DNA. The PCR primers M13: 5’-GTAAAACGACGGCCAGT-3’ and M13 reverse: 5’-AACAGCTATGACCATG-3’ were used for amplification of cDNA inserts in pOTB7 and pDNR-LIB plasmids. The PCR primers T3: 5’-AATTAACCCTCACTAAAGGG-3’ and T7: 5’-GTAATACGACTCACTATAGGGG-3’ were used for amplification of cDNA inserts in Bluescript® SK- plasmids. The PCR conditions were 94°C for 4 min, followed by 30 cycles at 94°C for 1 min, 55°C for 1 min, and 72°C for 1 min, with a final extension of 72°C for 5 min. PCR products were cleaned up by filtration using robotics stations (Multiscreen™ PCR, Millipore, USA; BioRobot 8000, Qiagen, Germany) and eluted in water.

High quality PCR (See Quality assessment workflow section) were pooled, then dried down. A total of 4 pmol of each product were re-suspended into 384-well plates and were combined 1:1 with DMSO to reach the printing criteria.

***Array printing*.** Microarrays were printed using the set of high-quality PCR products by high speed robotics with the 4^th^ generation Lucidea™ array spotter (GE Healthcare, USA) onto amino-modified mirrored glass slides: two hits per feature; one to six individual features per gene. Each array contains several prokaryotic and eukaryotic genes that serve as hybridization controls from RNA spikes added to experimental samples (Lucidea™ Universal Scorecard™, Lucidea™ microarray Scorecard™ v1.1, GE Healthcare, USA; and SpotReport™-3 array validation system, Stratagene, USA). This includes 11 housekeeping genes, 3 positive controls and 10 negative controls, consisting of *Human*, *Bacillus subtilis* or *Arabidopsis thaliana* genes, 10 calibration controls, 6 dynamic range controls and 8 ratio range controls. Spot size of each element is approximately 170 µm in diameter and spot centers 185 µm apart. Adhesion of the DNA to the glass was achieved by irradiation in the Hoefer model UVC500 illuminator (Hoefer, USA) with an energy output of 10^6^ µJ/cm². Microarrays were routinely stored in opaque plastic slide boxes at room temperature.

**Gene expression measurements**

***RNA purification.*** Frozen tissue samples specimens, in pieces from 50 to 150 mg each, were removed from the freezer, immediately placed into 700 µl of lysis Buffer RLT (Qiagen, Germany) together with stainless steel beads and homogenized using a Mixer Mill MM 300 (Qiagen, Germany), at 30 Hz over a period of 3 min. Then, homogenization was carried on as described in the commonly used RNeasy™ procedure, including an additional treatment step to remove residual DNA contamination with 30 units RNase-free DNase I.

***T7-linear amplification*.** One-round of RNA amplification was performed on total RNA based on a previously described protocol [11, 12] with some modifications. For first strand cDNA synthesis, 1 µg total RNA was annealed to 100 pmol T7-oligo-dT-primer (5’-ggCCAgTgAATTgTAATACgACTCACTATAgggAggCgg-(dT)24-3’) in RNase-free water (total volume 9 µl, 10 min, 70°C). The RNA/primer mixture was reverse transcribed in a total volume of 20 µl containing 4 µl 5x first stand buffer (Invitrogen, USA), 2 µl DTT, 1 µl dNTP Mix (10 mM) and 200 units Superscript™ II reverse transcriptase (Invitrogen, USA) at 37°C for 1 hr. The second-strand synthesis was performed at 16°C for 2 hr in a 150 µl reaction by mixing the first strand synthesis reaction in presence of *E. coli* enzymes, DNA polymerase I (40 units), DNA ligase (10 units) and RNase H (2 units) in 1x second strand buffer (Invitrogen, USA). The double-stranded cDNA (ds-cDNA) was blunt-ended using 20 units T4 DNA polymerase (Invitrogen, USA) for 5 min at 16°C. The reaction was stopped by the addition of 10 µl 0.5 M EDTA, pH 8.0 and products purified over QIAquick PCR purification columns (Qiagen, Germany) according to the manufacturer’s instructions, with minor modifications. The ds-cDNA was subsequently air dried in a vacuum centrifuge and the *in vitro* transcription (IVT) was carried out according to the manufacturer’s instructions (Ambion, USA) using the T7 MEGAscript™ kit. Two microliters of each 75 mM NTP, 2 µl of 10x reaction buffer and 2 µl of T7 polymerase enzyme mix was added with additional 200 units T7 RNA polymerase to a 4/10 volume of the ds-cDNA reaction. The remaining part (6/10) of the reaction was stored to perform quality assessment (See Quality assessment workflow Section). The IVT reaction was carried out at 37°C for 6 hr. Template DNA within the amplified antisense RNA (aRNA) was digested with 2 units DNase I at 37°C for 35 min, and cleaned up using RNeasy mini columns (Qiagen, USA) as described by the manufacturer. Amplified RNA products were air dried, resuspended in 10µl RNase-free water and then stored at -80°C; 1 µl of the reaction (1/10) was stored to perform quality assessment (See Quality assessment workflow Section).

***Targets labeling.*** Microarray targets were synthesized using 1.0 µg amplified RNA, 1 µl anchored oligo-(dT) and 2 µl random nonamers (GE Healthcare, USA) for priming (total volume 11 µl, 10 min, 70°C) in presence of 1 µl spike RNA from Lucidea™ Microarray Human Scorecard v1.1 (GE Healthcare, USA). cDNA products were generated using 1 mM of either Cy3- or Cy5-dCTP (GE Healthcare, USA) and 200 units MMLV RNase H- reverse transcriptase (Promega, USA) at 42°C for 2 to 3 hr. After reverse transcription, the RNA was degraded by adding 2 µl of 2.5 N NaOH and incubating at 37°C for 10 min. The reaction was then neutralized by adding 10 µl of 2 M MOPS (free acid) and Cy3- and Cy5-containing targets were purified twice onto QIAquick PCR columns (Qiagen, Germany) according the manufacturer’s instructions with minor modifications. The Cy-dye targets were controlled during quality assessment (See Quality assessment workflow Section).

Finally, an adjusted Cy-dye target yield was calculated to reflect carryover of unlabeled aRNA. Thus, certified targets were air-dried in a vacuum centrifuge for 1.5 hr and resuspended in RNase-free water to reach a 10 pmol/µl concentration.

***Hybridization.*** A mixture of equimolar aliquots (20 pmol) of each labeled cDNA (test and reference) was incubated at 95°C for 4 min in the presence of 1.5 µg Oligo-A_80_ (Eurogentec, USA) and 1 µg human Cot-I DNA® (Invitrogen, USA). Fragmented labeled cDNA was then placed onto 11K cDNA array slides in a 30 µl hybridization solution (50% formamide, GE Healthcare, USA) and covered by a coverslip. The slides were subsequently hybridized in a humid hybridization cabinet (GE Healthcare, USA) for 16 hr at 42°C. Then the hybridized slides were washed in 1xSSC, 0.2% SDS for 10 min at 55°C, twice in 0.1x SSC, 0.2% SDS for 10 min at 55°C and twice in 0.1x SSC for 1 min at room temperature. After washing and drying, the arrays were scanned at 532 nm (PMT 600 V) and 635 nm (PMT 650 V) using a 3^rd^ generation array scanner (GE Healthcare, USA) and independent grayscale images were generated for each pair of samples to be compared.

**Quality assessment workflow**

*Quality Controls of RNA samples.* Concentration and purity of the RNA preparation was checked first by UV-spectrophotometry using the Ultrospec3100 *pro* (GE Healthcare, USA) and 5-mm cuvettes. The absorbance (A) was measured from 200 nm to 350 nm. A_235_, A_260_, A_280_ and A_320_ were determined and A_260_:A_280_ ratios were calculated. Quality of each total RNA was measured by microfuidic/capillary electrophoresis using RNA 6000 Nano LabChip® kits with an Agilent 2100 Bioanalyzer (Agilent Technologies, USA), and the results assessed by human inspection and with two-user independent classifiers evaluation as described previously [98]. Only samples that reached the anticipated purity and integrity parameters (RIN >= 6; DegFact <= 13) were introduced within the microarray pipeline process.

(cf. Imbeaud et al. [98])

*Quality Controls of IVT transcription.* Double stranded cDNA (ds-cDNA) and amplified RNA (aRNA) products were analyzed for purity and concentration by UV spectrophotometry, using the Ultrospec3100 *pro* and 5-mm cuvettes (GE Healthcare, USA). The absorbance (A) was measured from 200 nm to 350 nm. A_235_, A_260_, A_280_ and A_320_ were determined and A_260_:A_280_ ratios were calculated. Three metrics were considered based on the A_260_ measurement. First, efficiency of the reverse transcription (ds-cDNA quantity/total RNA quantity) and IVT (aRNA quantity/ds-cDNA quantity) reaction efficiencies were computed. The yield of amplification was quantified (aRNA quantity/mRNA quantity), with the assumption that total RNA contains 2% of mRNA. Finally, an aliquot (1 µl) of the IVT reaction was loaded onto RNA 6000 Nano Labchip® using a Agilent 2100 Bioanalyzer (Agilent Technologies, USA). The chip was prepared according to the manufacturer’s protocol. An mRNA nano assay was run on the chip, and the size of the aRNA was compared with the molecular weight markers.

On one hand, ds-cDNA product with unsatisfactory reverse transcription reaction efficiency (RT ≤ 0.4) and purity (A_260_:A_280_ ratios ≤ 1.6) were removed from the IVT reaction pipeline. On the other hand, aRNA products with unsatisfactory yield of amplification (amplification < 1,000) and integrity (mean size < 1,000 nucleotides) were removed from subsequent analyses. Only samples that reached the expected purity and integrity parameters described above were introduced within the microarray labeling and hybridization pipelines.

*Quality Controls of Cy-Dye targets labeling.* Purity and concentration of each target was checked by UV spectrophotometry, using the Ultrospec3100 *pro* and 70-mm cuvettes (GE Healthcare). Based on the absorbance measured at 256 nm, 550 nm and 650 nm and considering extinction factor for Cy3- and Cy5-dye respectively of 0.15 and 0.25, the yield of cDNA synthesis and dye incorporation rate were computed. An aliquot (1 µl) of the purified labeling reaction was loaded onto RNA 6000 Nano Labchip® using a Agilent 2100 Bioanalyzer (Agilent Technologies, USA). The chip was prepared according to the manufacturer’s protocol and a mRNA nano assay was run on the chip. The shape and sizes of the Cy-dye targets were compared with the molecular weight markers. Targets with unsatisfactory yield of incorporation (dye incorporation rate < 10; A_256_:A_550 or 650_ < 2) and integrity (size < 200 nucleotides) were removed from subsequent analyses. The shape of the Cy-dye probes should present a smeared profile with no clearly identified additional peaks.

*Quality Controls of PCR probes synthesis.* Aliquots of each PCR product was analyzed for quality by UV measurement and agarose gel electrophoresis (< 2%) with ethidium bromide staining. Sample amplification is considered to have failed when multiple bands, unexpected product size or low integrity is observed. A low A_260_:A_280_ ratio is indicative of a low purity of the PCR product and may be associated with alcohol, detergent and protein contaminations, resulting in a high absorbance at 235 nm and 320 nm. These products were flagged out and specifically re-amplified as described above. A re-sequencing step was randomly performed for 689 clones distributed across the plates, in order to identify any manipulation errors and to allow refinement of the annotations. Representative PCR products were quantified at the Genome Express Service Center (France) using PicoGreen dyes (Molecular Probes, USA) in a fluorescent assay measuring double-strand DNA concentration according to the manufacturer’s instruction. Samples with quantity under 4 pmol, a threshold for our microarrays, are considered to have failed, were flagged out and then re-amplified.

**Statistical precision and power simulation**

The *statistical power* (Z; 1-ß) was computed to control that the number of individual patients is a reasonable compromise that guarantee sufficiently powered studies, allow a small number of spurious discoveries (α=0.003), while limiting the proportion of false negatives (ß). Calculation for two-sample comparison statistics considers individual biological replicates using the following formula:

where t_(1-α, df)_ is the inverse of the Student’s t cumulative distribution, 1-α being the probability of not getting false positives results and df the degree of freedom; δ is the effect size considering a true difference in mean expression levels between the two classes and observed biological variability (σ); and n_1_ and n_2_ the group sizes for clinical samples from individual patients displaying either a resistant or sensitive drug responses.

**REFERENCES**

52. M Ashburner, CA Ball, JA Blake, D Botstein, H Butler, JM Cherry, AP Davis, K Dolinski, SS Dwight, JT Eppig, et al: **Gene ontology: tool for the unification of biology. The Gene Ontology Consortium**. *Nat Genet* 2000, **25**:25-9.

95. RL Strausberg, EA Feingold, LH Grouse, JG Derge, RD Klausner, FS Collins, L Wagner, CM Shenmen, GD Schuler, SF Altschul, et al: **Generation and initial analysis of more than 15,000 full-length human and mouse cDNA sequences**. *Proc Natl Acad Sci U S A* 2002, **99**:16899-903.

98. S Imbeaud, E Graudens, V Boulanger, X Barlet, P Zaborski, E Eveno, O Mueller, A Schroeder, C Auffray: **Towards standardization of RNA quality assessment using user-independent classifiers of microcapillary electrophoresis traces**. *Nucleic Acids Res* 2005, **33**:e56.

100. J Eberwine, H Yeh, K Miyashiro, Y Cao, S Nair, R Finnell, M Zettel, P Coleman: **Analysis of gene expression in single live neurons**. *Proc Natl Acad Sci U S A* 1992, **89**:3010-4.

114. R Mariage-Samson, P Martinez, C Couillault, E Graudens, C Auffray, S Imbeaud, E Eveno: **Development of Quality Indices for cDNA microarray resources to improve gene expression profiling data interpretation**. In: *Transcriptome 2005 International Conference*; 2005; Shanghai, China.

115. DL Wheeler, DM Church, AE Lash, DD Leipe, TL Madden, JU Pontius, GD Schuler, LM Schriml, TA Tatusova, L Wagner, et al: **Database resources of the National Center for Biotechnology Information: 2002 update**. *Nucleic Acids Res* 2002, **30**:13-6.

116. GG Sutton, O White, MD Adams, AR Kerlavage: **TIGR Assembler: A new tool for assembling large shotgun sequencing projects**. *Genome Science and Technology* 1995, **1**:9-19.

117. EF Kirkness, AR Kerlavage: **The TIGR human cDNA database**. *Methods Mol Biol* 1997, **69**:261-8.

118. T Imanishi, T Itoh, Y Suzuki, C O'Donovan, S Fukuchi, KO Koyanagi, RA Barrero, T Tamura, Y Yamaguchi-Kabata, M Tanino, et al: **Integrative annotation of 21,037 human genes validated by full-length cDNA clones**. *PLoS Biol* 2004, **2**:856-75.

119. B Boeckmann, A Bairoch, R Apweiler, MC Blatter, A Estreicher, E Gasteiger, MJ Martin, K Michoud, C O'Donovan, I Phan, et al: **The SWISS-PROT protein knowledgebase and its supplement TrEMBL in 2003**. *Nucleic Acids Res* 2003, **31**:365-70.

120. J Kelso, J Visagie, G Theiler, A Christoffels, S Bardien, D Smedley, D Otgaar, G Greyling, CV Jongeneel, MI McCarthy, et al: **eVOC: a controlled vocabulary for unifying gene expression data**. *Genome Res* 2003, **13**:1222-30.

121. DA Benson, I Karsch-Mizrachi, DJ Lipman, J Ostell, DL Wheeler: **GenBank: update**. *Nucleic Acids Res* 2004, **32 Database issue**:D23-6.

122. RN Van Gelder, ME von Zastrow, A Yool, WC Dement, JD Barchas, JH Eberwine: **Amplified RNA synthesized from limited quantities of heterogeneous cDNA**. *Proc Natl Acad Sci U S A* 1990, **87**:1663-7.
