# Supplementary material for: Deciphering cellular states of innate tumor drug responses
Source: Genome Biol. 2006 Mar 15;7(3):R19. doi: 10.1186/gb-2006-7-3-r19 (PMC1557757; doi:10.1186/gb-2006-7-3-r19)
Supplement: Additional data file 6 — The gene expression matrix of the significant node NODE547X. [file gb-2006-7-3-r19-S6.doc]

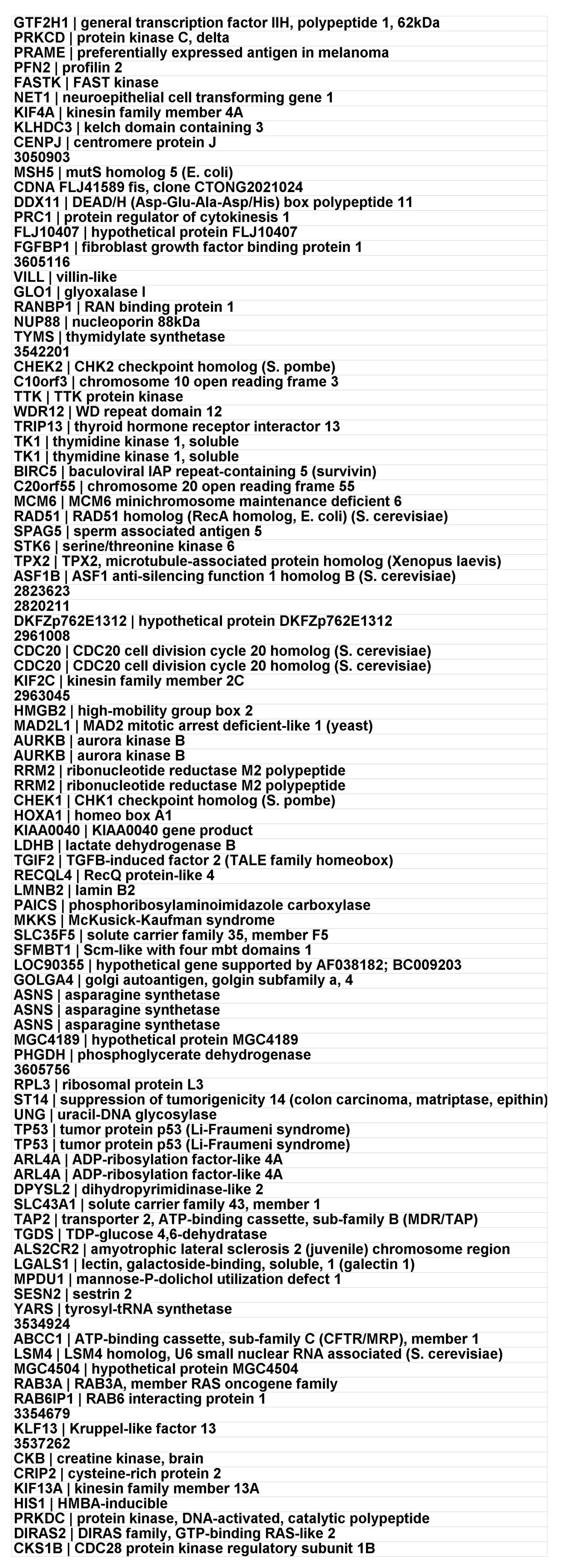

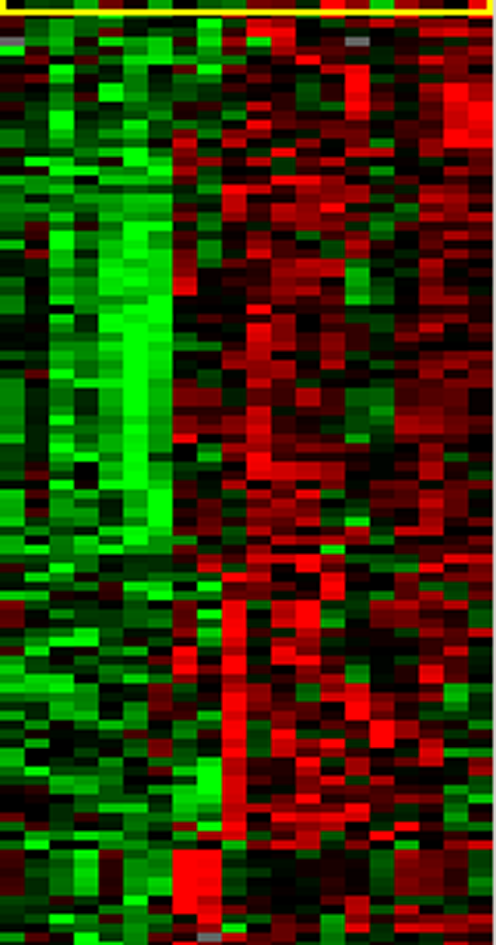


***p*-value ≤ 6.00e-05**

**Additional Data File 6: Top-ranked discriminating cluster – NODE547X**

Matrix representation of expression levels from NODE547X, in which 102 elements are up-regulated in resistant state samples. Color bars refer to the location of the gene cluster (left) and subsequent patient drug response rate (top) as displayed in Figure 2 of the manuscript. The genes are referenced by gene symbol followed by the gene name (for a gene with no identifier, the I.M.A.G.E. Clone ID is indicated) according to the international nomenclature.
