# Supplementary material for: Deciphering cellular states of innate tumor drug responses
Source: Genome Biol. 2006 Mar 15;7(3):R19. doi: 10.1186/gb-2006-7-3-r19 (PMC1557757; doi:10.1186/gb-2006-7-3-r19)
Supplement: Additional data file 7 — A serial representation of the relative expression of a series of genes collected by Q-PCR during the validation step performed on a new metastasis sample collected from an additional patient. [file gb-2006-7-3-r19-S7.doc]

**Additional Data File 7: Validation of the microarray gene expression data on new cancer samples**

Serial representation of Q-PCR relative expression (2^-ΔΔCt^; log_10_) of a series of 18 genes (Symbol - HUGO nomenclature) that were shown to be statistically differentially expressed in both microarray and Q-PCR (indicated in gray, cf. Table 1 in the manuscript).

Results are displayed as circles (○) for the mean 2^-ΔΔCt^ and 95% confidence intervals computed (as described in Materials & Methods section of the manuscript) on a series of cancerous samples, which correspond to the sample set previously used in the microarray analysis (cf. Additional Data File 10 online). The color patch refers to the primary drug responses, either chemo-sensitive (in blue) or resistant (in red), of the corresponding patients.

Squares (□) show the relative expression levels measured on a new metastasis sample (M-P52) from an additional patient (P52). The observed expressions were found mostly predictive of a chemo-sensitive state, in agreement with the fact that the patient P52 was subsequently diagnosed as sensitive at the presentation of the drugs.
